# Nonmotor Symptom Changes and Their Association With Falls Among Parkinson's Disease Patients Undergoing Deep Brain Stimulation: A 1‐Year Cohort Study

**DOI:** 10.1111/cns.70310

**Published:** 2025-02-28

**Authors:** Ying Gao, Hui You, Jue Wang, Mengsi Yao, Dianyou Li, Bomin Sun, Linbin Wang, Xian Qiu

**Affiliations:** ^1^ Department of Nursing Ruijin Hospital, Shanghai Jiao Tong University School of Medicine Shanghai China; ^2^ School of Nursing, Shanghai Jiao Tong University School of Medicine Shanghai China; ^3^ Department of Neurosurgery Ruijin Hospital, Shanghai Jiao Tong University School of Medicine Shanghai China; ^4^ Department of Psychiatry University of Cambridge Cambridge UK

**Keywords:** deep brain stimulation, falls, generalized estimating equations, nonmotor symptoms, Parkinson's disease

## Abstract

**Objectives:**

Fall severely affects the quality of life of Parkinson's disease (PD) patients. Subthalamic nucleus (STN) deep brain stimulation (DBS) is an effective treatment for PD motor symptoms (MS), but DBS increased the risk of falls in some studies and has mixed effects on nonmotor symptoms (NMS). However, the link between NMS and falls, and how DBS influences this relationship, remain unclear. This study investigated changes in NMS and falls before and after STN‐DBS, and the longitudinal association between NMS and falls.

**Methods:**

The study included 136 PD patients undergoing STN‐DBS between April 2020 and February 2022. Data were collected preoperatively, at 6 months, and at 12 months postoperatively. Assessments included MS via the Unified Parkinson's Disease Rating Scale‐III (UPDRS‐III) and NMS via the Nonmotor Symptoms Scale (NMSS). We used the Friedman and chi‐square tests to assess changes in NMS and falls. Specific circumstances of falls were assessed through structured interviews. Generalized estimating equations (GEE) were used to explore the longitudinal associations between NMS and fall occurrence, as well as the interaction effects between MS and NMS on fall occurrence.

**Results:**

Significant improvements (*p* < 0.01) were observed in all NMSS domains except gastrointestinal, with no change in fall occurrence. However, there were significant changes in both the locations where falls occurred and whether freezing of gait was present among falling patients (*p* < 0.01). GEE analysis revealed significant associations between falls and mood/cognition (*p* = 0.044), gastrointestinal (*p* = 0.027), and urinary symptoms (*p* = 0.007), as well as interactions between motor and these NMS domains (*p* < 0.05).

**Conclusions:**

NMS, particularly mood/cognition, gastrointestinal, and urinary symptoms, and their interactions with MS, are associated with falls, underscoring the need for targeted fall prevention strategies.

## Graphical abstract

It is the first study to link nonmotor symptoms with falls in Parkinson's patients post‐DBS. Generalized estimating equations were used to assess the longitudinal impact of NMS with falls in 136 patients, finding that mood/cognition, gastrointestinal, and urinary symptoms, as well as their interactions with motor symptoms, were related to fall risk.

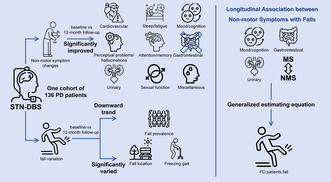

## 1 Introduction

Parkinson's disease (PD) is the second most common neurodegenerative disorder, characterized by the degeneration of dopaminergic neurons in the substantia nigra [1, 2]. It presents with motor symptoms (MS) such as tremors, rigidity, bradykinesia, and postural instability, as well as nonmotor symptoms (NMS) such as mood disorders, cognitive disability, sleep disturbances, and urinary issues [3, 4]. PD affects 1–2 individuals per 1000 people globally [5], and its prevalence is rising with the aging population [6]. By 2030, China alone could have 4.94 million PD patients, making up over half of the global PD population [7].

Falls are a significant concern for PD patients, substantially reducing their quality of life. Each year, 60.5% of PD patients experience falls, and among them, two‐thirds suffer from recurrent falls—a rate twice that of the general elderly population [8, 9, 10]. Frequent falls result in elevated rates of injury and disability. In a study of over 1000 PD patients, 65% had injuries from falls, and 33% suffered fractures [11], placing a heavy burden on families and healthcare systems. The variability in fall situations complicates prevention, highlighting the need for tailored interventions.

PD results from a complex interplay of MS, NMS, medication effects, and environmental factors [12]. While motor impairments such as postural instability and gait disturbances are well‐established contributors to falls, emerging evidence underscores the critical role of NMS. Orthostatic hypotension, mood disturbances (e.g., anxiety, depression), and cognitive deficits (e.g., impaired attention, executive dysfunction) have been increasingly recognized as independent risk factors for falls in PD [13, 14]. Despite this, the mechanistic relationship between NMS and falls—particularly in the context of therapeutic interventions like subthalamic nucleus deep brain stimulation (STN‐DBS)—remains poorly understood.

STN‐DBS is a cornerstone surgical therapy for advanced PD, offering robust improvements in MS and reducing dopaminergic medication burden [15, 16]. However, its effects on falls are paradoxical: while motor function may improve, studies report an increased risk of falls post‐DBS, potentially linked to residual gait instability or stimulation‐induced side effects [17]. Furthermore, the impact of DBS on NMS is inconsistent, with evidence suggesting both improvements (e.g., sleep) and worsening (e.g., apathy, cognitive decline) across domains [18, 19, 20, 21]. Critically, no study has systematically examined whether longitudinal changes in NMS after DBS mediate the observed increase in fall risk, leaving a pivotal gap in understanding how to optimize postsurgical outcomes [22].

To address this gap, we enrolled 277 PD patients undergoing STN‐DBS and assessed their falls and NMS before surgery, as well as at 6 and 12 months postoperatively. We leveraged mixed quantitative and qualitative methods and data‐driven analysis, aiming to: (1) investigate changes in NMS and falls before and after STN‐DBS, and (2) explore the longitudinal association between NMS and fall risk across the study period.

## 2 Materials and Methods

### 2.1 Patients and Study Design

This cohort study focuses on 277 PD patients who underwent STN‐DBS. Patients were recruited from April 2020 to February 2022 at the Department of Functional Neurosurgery, Ruijin Hospital, Shanghai, China. A total of 136 patients who met inclusion criteria and completed 6‐ and 12‐month follow‐ups were included. A total of 144 patients were excluded due to incomplete follow‐up or partially missing follow‐up data, ensuring the integrity and completeness of the final dataset.

Inclusion criteria were: (1) PD diagnosis, (2) undergoing STN‐DBS treatment, and (3) age ≥ 18 years. Exclusion criteria were: (1) conditions deemed by neurologists to hinder follow‐up (e.g., cognitive impairment, organic neurological disorders, communication difficulties), (2) drug abuse, (3) surgical complications above grade I [23], and (4) incomplete data.

Data collection involved clinical assessments and questionnaire surveys conducted preoperatively, as well as at 6 and 12 months postoperatively. The study has been approved by the Ethics Committee of Ruijin Hospital (Ruijin Hospital Ethics Committee [2023] No. 122).

### 2.2 Outcome Measures

NMS were evaluated using the Nonmotor Symptoms Scale (NMSS), covering nine domains including cardiovascular, sleep/fatigue, mood/cognition, perceptual problems/hallucinations, attention/memory, gastrointestinal, urinary, sexual, and miscellaneous symptoms. Items were rated based on frequency (1–4) and severity (0–3), with higher scores indicating greater severity. The NMSS has demonstrated strong internal consistency (Cronbach's alpha: 0.89) [24]. MS were assessed using the Unified Parkinson's Disease Rating Scale Part III (UPDRS‐III), with scores ranging from 0 to 108; higher scores indicate more severe symptoms [25].

A fall was characterized as any incident involving a slip, trip, or loss of balance that resulted in the participant making unintended land with the floor, ground, or a lower surface [26, 27]. We developed a structured interview instrument for fall risk assessment and fall classification according to a previous review [28] and suggestions from experts specializing in neurology. The interview was structured based on a list of simple questions to elicit information concerning the presence of a fall and details about the fall. Particularly, falls were self‐reported as a binary variable: 1 for a fall in the past month, and 0 for no falls. The PD patient fall questionnaire and additional information including fall frequency, fall location, and so on were recorded and are detailed in Supporting Information.

Sociodemographic and disease‐related factors known to influence PD were controlled. These factors included age (< 60 or ≥ 60 years), gender, residence (Rural or Urban), education level (Junior high school and below, High school, or Associate degree and above), disease duration, MS assessed by the UPDRS‐III, Hoehn–Yahr (H‐Y) stage, PD subtypes (PIGD, TD, or Intermediate), comorbidities, levodopa equivalent daily dose (LEDD) [29], body mass index (BMI), and hemoglobin (Hb).

NMS, LEDD, and falls were assessed presurgery and at 6 and 12 months postsurgery. All information was collected or evaluated in the medication‐off state.

### 2.3 Data Analysis

All statistical analyses were performed using IBM SPSS version 22.0, with a significance level set at 0.05. The normality of data distribution was assessed using the Shapiro–Wilk test. Continuous variables were presented as mean (standard deviation, SD) or median (interquartile range, IQR), while categorical variables were expressed as frequencies (%). Differences in fall occurrence and NMSS scores across stages were evaluated using the chi‐square test and Friedman test, respectively. Violin plots were also displayed for clearer visualization. Post hoc comparisons were adjusted using the Bonferroni correction method.

We performed subgroup analyses to explore differences between falling and nonfalling patients at baseline, 6 months, and 12 months postoperatively, as well as subgroup comparisons between postoperative fallers and nonfallers. Categorical variables were analyzed using the chi‐square test, while continuous variables were assessed using the Mann–Whitney *U* test or the independent samples *t*‐test.

Generalized estimating equations (GEEs) were used to analyze the longitudinal associations between NMS and fall occurrence, as well as the interaction effects between MS and NMS on fall occurrence. The models were adjusted for time‐constant, time‐varying, and time‐fixed covariates [30]. The dependent variable was fall occurrence, assessed at baseline, 6‐month, and 12‐month follow‐ups. Main factors included demographic data, disease duration, UPDRS‐III, H‐Y stage, PD subtype, LEDD, hemoglobin, and NMSS, with interaction effects focusing on UPDRS III and various NMSS domains.

## 3 Results

### 3.1 Demographic and Disease‐Related Information

A total of 136 patients diagnosed with PD were enrolled in this study. The average age of the participants was 62.8 years (SD = 9.5). Among them, 80 individuals (58.8%) were male. Besides, the median disease duration was 8.0 years (IQR = 5.0), with a median LEDD of 700.0 mg per day (IQR = 426.0). The average UPDRS‐III score was 57.8 (SD = 13.2). Other demographic and disease information is shown in Table S1.

### 3.2 Changes in Fall Occurrence and Nonmotor Symptoms

**FIGURE 1:**
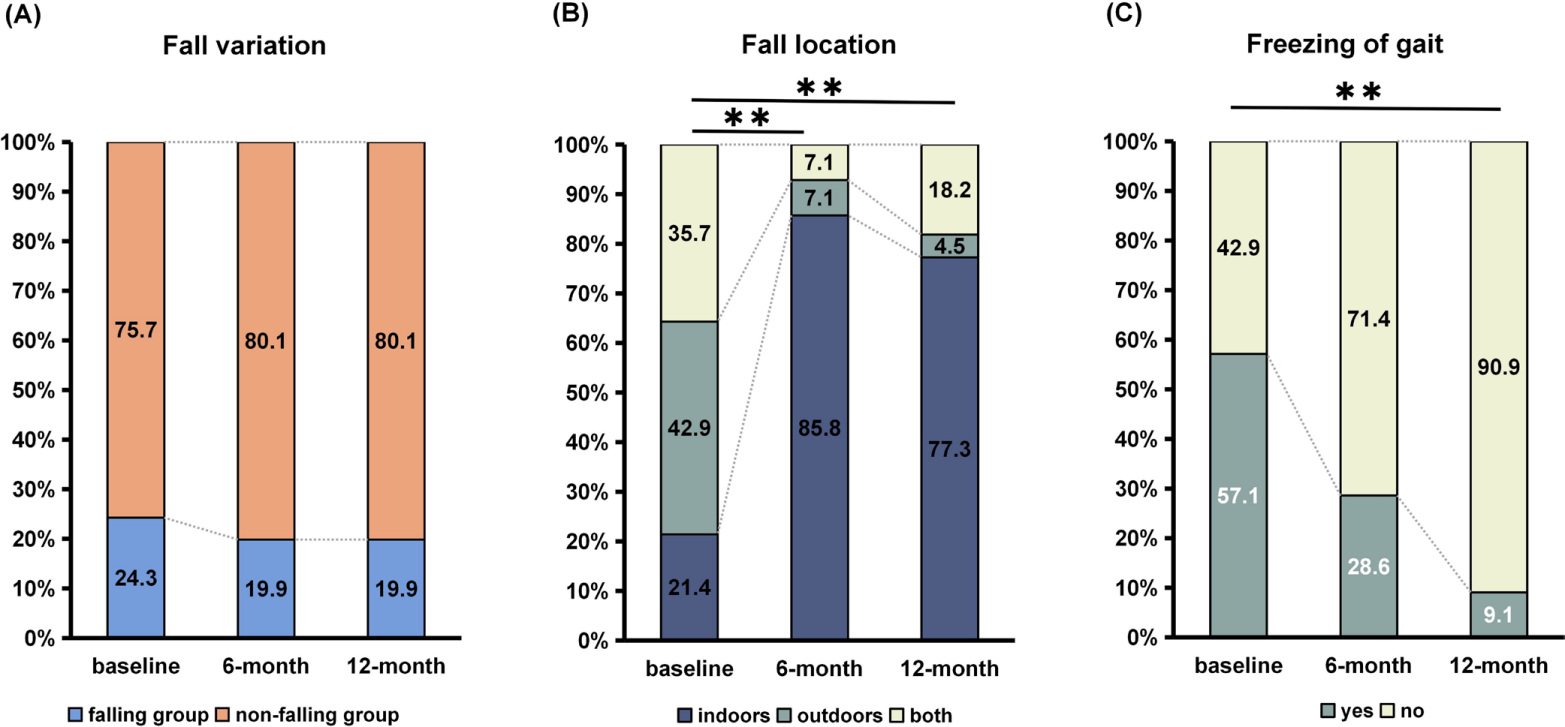
Changes in (A) fall variation, (B) fall locations, and (C) freezing of gait between baseline, 6‐month follow‐up, and 12‐month follow‐up. **means *p* < 0.01.

Preoperatively, 33 patients (24.3%) experienced falls in the past month, while 27 (19.9%) experienced falls at the 6‐month and 27 (19.9%) at the 12‐month follow‐ups. There was no significant change when comparing preoperative and postoperative states (*χ* ^2^ = 1.052, *p* = 0.641) (Figure 1). Of the 33 patients who fell before surgery, 19 (57.6%) did not fall after STN‐DBS. Conversely, among the 103 patients who did not fall before surgery, 26 (25.2%) experienced falls post‐surgery.

Significant differences were observed in the fall locations and the presence of freezing of gait between baseline and follow‐up visits. Before STN‐DBS, falls were relatively balanced between indoor, outdoor, and both locations (21.4%, 42.9%, and 35.7%, respectively), while the majority of falls occurred indoors postoperatively, accounting for 85.8% at 6 months (*p* = 0.002) and 77.3% at 12 months (*p* = 0.001). The presence of freezing of gait in falling patients also showed significant changes (*p* = 0.003). Preoperatively, 57.1% of falling patients had freezing of gait, while at 12 months, only 9.1% exhibited freezing of gait. Further details on the circumstances of falls are presented in Table S2 and Figure S1.

**FIGURE 2:**
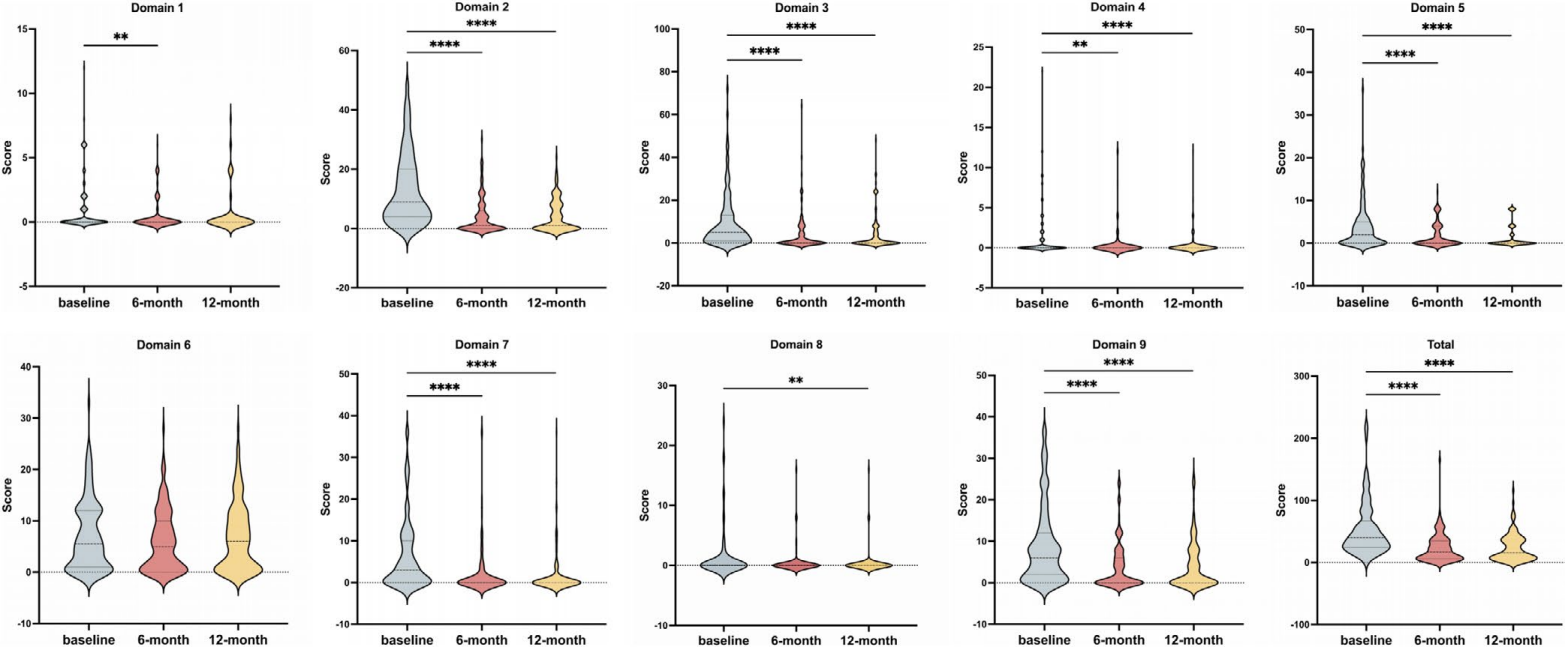
Violin plots displaying post hoc analysis results for NMSS scores in PD patients preoperatively, 6 months postoperatively, and 12 months postoperatively. **means *p*′ < 0.01, ***means *p*′ < 0.001, and ****means *p*′ < 0.0001.

We compared NMSS scores across nine domains between baseline and follow‐up visits to evaluate the impact of STN‐DBS on NMS. The results revealed significant improvement in total NMSS scores at 6‐ and 12‐month follow‐ups compared to preoperative states (Table 1). All NMSS domains significantly improved with the exception of the gastrointestinal domain (Figure 2).

**TABLE 1:** Longitudinal changes in NMSS scores preoperatively, 6 months postoperatively, and 12 months postoperatively in PD patients.

	Maximum score	Baseline	6‐month follow‐up	12‐month follow‐up	*z*	*p*
NMSS domains						
NMSS total score	360.0	52.9 (43.3)	23.2 (22.7)	23.5 (21.7)	83.732	**< 0.001**
NMSS domain 1: Cardiovascular	24.0	1.0 (2.1)	0.5 (1.2)	0.6 (1.7)	12.604	**0.002**
NMSS domain 2: Sleep/fatigue	48.0	13.1 (11.8)	4.1 (6.1)	4.2 (5.4)	91.515	**< 0.001**
NMSS domain 3: Mood/cognition	72.0	9.8 (12.8)	4.2 (8.6)	4.2 (8.2)	46.248	**< 0.001**
NMSS domain 4: Perceptual problems/hallucinations	36.0	1.2 (3.0)	0.4 (1.7)	0.3 (1.3)	31.829	**< 0.001**
NMSS domain 5: Attention/memory	36.0	4.0 (5.6)	1.8 (2.8)	1.4 (2.6)	37.132	**< 0.001**
NMSS domain 6: Gastrointestinal	36.0	7.1 (6.8)	6.0 (6.0)	6.7 (6.5)	4.552	0.101
NMSS domain 7: Urinary	36.0	6.3 (8.5)	1.9 (5.5)	1.5 (2.8)	83.883	**< 0.001**
NMSS domain 8: Sexual function	24.0	1.5 (4.4)	0.6 (2.4)	0.5 (2.3)	18.242	**< 0.001**
NMSS domain 9: Miscellaneous	48.0	8.8 (9.3)	3.8 (5.6)	3.9 (5.8)	56.043	**< 0.001**

*Note:* Data are presented as mean (SD) due to the ambiguity associated with median (IQR) in this case. Friedman test was used to compare the longitudinal changes. Bold values mean *p* < 0.05.

Abbreviation: NMSS, Nonmotor symptoms scale.

### 3.3 Subgroup Analysis of Fallers and Nonfallers Post Surgery

We performed subgroup analyses to investigate differences between falling and nonfalling patients at baseline, 6 months, and 12 months postoperatively (Table 2). At baseline, only the urinary domain showed a significant difference between the two groups. At 6 months postoperatively, no significant differences were found in NMSS domains. By 12 months, significant differences emerged in the sleep/fatigue, attention/memory, gastrointestinal, and urinary domains, as well as in the total NMSS score, with the falling group having higher scores. Additionally, significant differences between the falling and nonfalling groups were found in residence, education, and LEDD by 12 months.

**TABLE 2:** Comparison of NMSS scores and disease information between falling and nonfalling PD patients preoperatively, and at 6 and 12 months postoperatively.

	Baseline				6‐month follow‐up				12‐month follow‐up			
	Nonfalling group (*N* = 103)	Falling group (*N* = 33)	*χ* ^2^/*z*/*t*	*p. adjust*	Nonfalling group (*N* = 109)	Falling group (*N* = 27)	*χ* ^2^/*z*/*t*	*p. adjust*	Nonfalling group (*N* = 109)	Falling group (*N* = 27)	*χ* ^2^/*z*/*t*	*p. adjust*
Age			2.962	0.126^a^			3.761	0.062^a^			2.163	0.166^a^
Age < 60	35 (34.0)	6 (18.2)			37 (33.9)	4 (14.8)			36 (33.0)	5 (18.5)		
Age ≥ 60	68 (66.0)	27 (81.8)			72 (66.1)	23 (85.2)			73 (67.0)	22 (81.5)		
Gender			0.329	0.685^a^			0.149	0.828^a^			0.856	0.391^a^
Male	62 (60.2)	18 (54.5)			65 (59.6)	15 (55.6)			62 (56.9)	18 (66.7)		
Female	41 (39.8)	15 (45.5)			44 (40.4)	12 (44.4)			47 (43.1)	9 (33.3)		
Residence			0.124	0.800^a^			5.176	**0.027** ^a^			5.176	**0.027** ^a^
Rural areas	19 (18.4)	7 (21.2)			25 (22.9)	1 (3.7)			25 (22.9)	1 (3.7)		
Urban areas	84 (81.6)	26 (78.8)			84 (77.1)	26 (96.3)			84 (77.1)	26 (96.3)		
Education			2.870	0.768^a^			4.933	0.252^a^			10.160	**0.015** ^a^
Junior high school and below	44 (42.7)	17 (51.5)			54 (49.5)	7 (25.9)			55 (50.5)	6 (22.2)		
High school	31 (30.1)	5 (15.2)			26 (23.9)	10 (37.0)			29 (26.6)	7 (25.9)		
Associate degree and above	28 (27.2)	11 (33.3)			29 (26.6)	10 (37.0)			25 (22.9)	14 (51.9)		
Disease duration, year	8.0 (5.0)	9.0 (7.5)	−1.181	0.239^b^	8.0 (5.0)	9.0 (8.0)	−1.171	0.244^b^	8.0 (5.0)	8.0 (8.0)	−0.063	0.951^b^
UPDRS‐III	57.1 (13.7)	60.1 (11.4)	−1.120	0.265^c^	57.1 (13.6)	60.9 (11.3)	−1.335	0.184^c^	57.6 (13.0)	58.6 (14.5)	−0.356	0.723^c^
H‐Y stage			0.821	> 0.999^a^			9.562	0.132^a^			3.962	0.795^a^
2.0	13 (12.6)	4 (12.1)			17 (15.6)	0 (0.0)			15 (13.8)	2 (7.4)		
2.5	35 (34.0)	9 (27.3)			38 (34.9)	6 (22.2)			36 (33.0)	8 (29.6)		
3.0	44 (42.7)	15 (45.5)			44 (40.4)	15 (55.6)			48 (44.0)	11 (40.7)		
4.0	11 (10.7)	5 (15.2)			10 (9.2)	6 (22.2)			10 (9.2)	6 (22.2)		
Subtype			5.409	0.219^a^			1.928	> 0.999^a^			0.669	> 0.999^a^
PIGD	62 (60.2)	23 (69.7)			65 (59.6)	20 (74.1)			68 (62.4)	17 (63.0)		
TD	26 (25.2)	10 (30.3)			31 (28.4)	5 (18.5)			30 (27.5)	6 (22.2)		
Intermediate	15 (14.6)	0 (0.0)			13 (11.9)	2 (7.4)			11 (10.1)	4 (14.8)		
With comorbidity			0.329	0.651^a^			0.815	0.465^a^			0.815	0.465^a^
No	77 (74.8)	23 (69.7)			82 (75.2)	18 (66.7)			82 (75.2)	18 (66.7)		
Yes	26 (25.2)	10 (30.3)			27 (24.8)	9 (33.3)			27 (24.8)	9 (33.3)		
LEDD at the corresponding follow‐up point, mg/day	700.0 (425.0)	700.0 (393.8)	−0.137	0.892^b^	350.0 (181.3)	350.0 (300.0)	−0.161	0.874^b^	300.0 (212.5)	400.0 (300.0)	−2.781	**0.005** ^b^
BMI, kg/m^2^			5.212	0.225^a^			3.164	0.660^a^			2.562	0.909^a^
BMI < 18.5	4 (3.9)	5 (15.2)			8 (7.3)	1 (3.7)			6 (5.5)	3 (11.1)		
18.5 ≤ BMI < 24.0	50 (48.5)	15 (45.5)			48 (44.0)	17 (63.0)			50 (45.9)	15 (55.6)		
BMI ≥ 24.0	48 (47.6)	13 (39.4)			53 (48.6)	9 (33.3)			53 (48.6)	9 (33.3)		
Hb, g/L			3.607	0.092^a^			5.258	0.053^a^			5.258	0.053^a^
Anemic	2 (1.9)	3 (9.1)			2 (1.8)	3 (11.1)			2 (1.8)	3 (11.1)		
Not anemic	101 (98.1)	30 (90.9)			107 (98.2)	24 (88.9)			107 (98.2)	24 (88.9)		
NMSS domains at the corresponding follow‐up point												
NMSS total score	50.4 (40.8)	60.6 (50.5)	−1.117	0.266^b^	21.0 (19.1)	32.0 (32.4)	−1.799	0.072^b^	19.3 (17.7)	40.3 (27.5)	−4.073	**< 0.001** ^b^
NMSS domain 1: Cardiovascular	0.9 (2.0)	1.5 (2.2)	−1.912	0.056^b^	0.4 (1.1)	0.7 (1.2)	−1.906	0.068^b^	0.5 (1.4)	1.1 (2.4)	−1.285	0.152^b^
NMSS domain 2: Sleep/fatigue	13.4 (12.1)	12.1 (11.0)	−0.320	0.751^b^	3.5 (5.1)	6.7 (8.5)	−1.416	0.158^b^	3.5 (4.6)	7.2 (7.0)	−2.704	**0.006** ^b^
NMSS domain 3: Mood/cognition	9.5 (13.0)	10.6 (12.6)	−0.312	0.757^b^	3.5 (6.9)	6.9 (13.3)	−1.197	0.234^b^	3.0 (6.2)	8.9 (12.6)	−2.746	0.475^b^
NMSS domain 4: Perceptual problems/hallucinations	1.1 (2.6)	1.5 (4.1)	−0.255	0.802^b^	0.2 (0.7)	1.3 (3.5)	−1.850	0.079^b^	0.2 (0.8)	0.7 (2.6)	−0.560	0.455^b^
NMSS domain 5: Attention/memory	3.6 (5.3)	5.3 (6.4)	−1.154	0.250^b^	1.7 (2.6)	2.2 (3.3)	−1.000	0.320^b^	1.3 (2.4)	1.9 (3.2)	−0.760	**0.016** ^b^
NMSS domain 6: Gastrointestinal	6.7 (6.7)	8.3 (7.1)	−1.252	0.212^b^	5.7 (6.0)	7.2 (5.1)	−1.675	0.094^b^	6.0 (6.1)	9.6 (7.3)	−2.398	**0.008** ^b^
NMSS domain 7: Urinary	5.6 (8.3)	8.5 (8.9)	−2.577	**0.010** ^b^	1.8 (5.1)	2.5 (7.2)	−0.921	0.380^b^	0.9 (3.3)	4.0 (8.2)	−2.469	**0.008** ^b^
NMSS domain 8: Sexual function	1.1 (3.4)	2.9 (6.7)	−0.625	0.548^b^	0.7 (2.7)	0.0 (0.0)	−1.628	0.185^b^	0.3 (1.9)	1.2 (3.7)	−1582	0.083^b^
NMSS domain 9: Miscellaneous	8.5 (9.3)	9.9 (9.4)	−0.998	0.321^b^	3.7 (5.4)	4.4 (6.4)	−0.454	0.653^b^	3.5 (5.5)	5.6 (6.8)	−1.611	0.108^b^

*Note:* All the information was collected during “off” conditions of patients. Data are presented as mean (SD) or median (IQR) for continuous variables and number (%) for categorical variables. NMSS data are presented as mean (SD) due to the ambiguity associated with median (IQR) in this case. Bold values mean adjusted *p* < 0.05 (Bonferroni correction).

Abbreviations: BMI, body mass index; Hb, hemoglobin; H‐Y stage, Hoehn–Yahr stage; LEDD, levodopa equivalent daily doses; NMSS, nonmotor symptoms scale; PIGD, postural instability/gait difficulty subtype; TD, tremor‐dominant subtype; UPDRS‐III, unified Parkinson's disease rating scale part III.

^a^ Chi‐square test.

^b^ Mann–Whitney U test.

^c^ Independent samples *t*‐test.

To identify latent predictors of falls after STN‐DBS, we also performed a subgroup analysis comparing postoperative fallers and nonfallers. The faller group included patients who experienced at least one fall during follow‐up, while the nonfaller group consisted of those without any falls post‐STN‐DBS. Significant differences were found in age, residence, education, and baseline urinary NMSS domain (Table 3).

**TABLE 3:** Comparison of baseline NMSS scores and disease information between postoperative falling and nonfalling PD patients.

	Postoperative nonfalling group (*N* = 96)	Postoperative falling group (*N* = 40)	*χ* ^2^/*z*/*t*	*p. adjust*
Age				
Age < 60	35 (36.5)	6 (15.0)	6.174	**0.014** ^a^
Age ≥ 60	61 (63.5)	34 (85.0)		
Gender				
Male	56 (58.3)	24 (60.0)	0.032	0.857^a^
Female	40 (41.7)	16 (40.0)		
Residence				
Rural areas	25 (26.0)	1 (2.5)	10.120	**0.001** ^a^
Urban areas	71 (74.0)	39 (97.5)		
Education				
Junior high school and below	51 (53.1)	10 (25.0)	9.534	**0.027** ^a^
High school	23 (24.0)	13 (32.5)		
Associate degree and above	22 (22.9)	17 (42.5)		
Disease duration, year	8.0 (4.8)	8.5 (7.5)	−1.097	0.273^b^
UPDRS‐III	57.3 (13.4)	59.0 (12.8)	0.672	0.503^c^
H‐Y				
2.0	15 (15.6)	2 (5.0)	6.167	0.624^a^
2.5	34 (35.4)	10 (25.0)		
3.0	38 (39.6)	21 (52.5)		
4.0	9 (9.4)	7 (17.5)		
Subtype				
PIGD	58 (60.4)	27 (67.5)	1.234	> 0.999^a^
TD	28 (29.2)	8 (20.0)		
Intermediate	10 (10.4)	5 (12.5)		
With comorbidity				
No	74 (77.1)	26 (65.0)	2.118	0.200^a^
Yes	22 (22.9)	14 (35.0)		
LEDD_baseline_, mg/day	675.0 (434.4)	787.5 (474.5)	−1.854	0.064^b^
BMI, kg/m^2^				
BMI < 18.5	5 (5.2)	3 (7.5)	2.585	0.879^a^
18.5 ≤ BMI < 24.0	43 (44.8)	23 (57.5)		
BMI ≥ 24.0	48 (50.0)	14 (35.0)		
Hb, g/L				
Anemic	1 (1.0)	3 (7.5)	4.126	0.076^a^
Not anemic	95 (99.0)	37 (92.5)		
NMSS domains_baseline_				
NMSS total score_baseline_	51.9 (43.7)	55.2 (43.0)	−0.597	0.550^b^
NMSS domain 1_baseline_: Cardiovascular	1.2 (2.2)	0.8 (1.5)	−0.141	0.888^b^
NMSS domain 2_baseline_: Sleep/fatigue	13.0 (11.8)	13.4 (11.7)	−0.251	0.802^b^
NMSS domain 3_baseline_: Mood/cognition	10.1 (13.9)	9.0 (9.9)	−0.079	0.937^b^
NMSS domain 4_baseline_: Perceptual problems/hallucinations	1.3 (3.1)	1.1 (2.7)	−0.966	0.334^b^
NMSS domain 5_baseline_: Attention/memory	4.0 (5.7)	4.0 (5.4)	−0.360	0.719^b^
NMSS domain 6_baseline_: Gastrointestinal	6.4 (5.9)	8.7 (8.4)	−1.103	0.270^b^
NMSS domain 7_baseline_: Urinary	5.4 (7.7)	8.5 (10.1)	−1.978	**0.048** ^b^
NMSS domain 8_baseline_: Sexual function	1.8 (4.8)	0.9 (3.4)	−1.288	0.198^b^
NMSS domain 9_baseline_: Miscellaneous	8.7 (8.8)	9.0 (10.6)	−0.408	0.683^b^

*Note:* Postoperative falling group: At least one fall occurred during the two postoperative visits; Postoperative nonfalling group: No fall occurred during the two follow‐up visits. All the information was collected during “off” conditions of patients. Data are presented as mean (SD) or median (IQR) for continuous variables and number (%) for categorical variables. NMSS data are presented as mean (SD) due to the ambiguity associated with median (IQR) in this case. Bold values mean adjusted *p* < 0.05 (Bonferroni correction).

Abbreviations: BMI, body mass index; Hb, hemoglobin; H‐Y stage, Hoehn–Yahr stage; LEDD, levodopa equivalent daily doses; NMSS, nonmotor symptoms scale; PIGD, postural instability/gait difficulty subtype; TD, tremor‐dominant subtype; UPDRS‐III, unified Parkinson's disease rating scale part III.

^a^ Chi‐square test.

^b^ Mann–Whitney *U* test.

^c^ Independent samples *t*‐test.

### 3.4 Nonmotor Symptoms Associated With Falls

Univariate GEE analysis revealed statistically significant differences in age, education, disease duration, H‐Y staging, LEDD, anemia status, and NMSS domains 1–7 (*p* < 0.05) (Table S3). The variables selected from the univariate GEE analysis, UPDRS‐III score, and subtype were then used as independent variables, while fall occurrence was used as the dependent variable in a multivariate GEE analysis (Table 4). The results showed that associate degree and above (Wald *χ* ^2^ = 11.200, *p* = 0.001), disease duration (Wald *χ* ^2^ = 4.512, *p* = 0.034), without anemia (Wald *χ* ^2^ = 7.667, *p* = 0.006), milder mood/cognition symptoms (Wald *χ* ^2^ = 4.063, *p* = 0.044), more severe gastrointestinal symptoms (Wald *χ* ^2^ = 4.893, *p* = 0.027), and more severe urinary symptoms (Wald *χ* ^2^ = 7.286, *p* = 0.007) correlated with the occurrence of falls.

**TABLE 4:** Multivariate generalized estimating equation analysis of factors associated with falls after STN‐DBS in PD patients.

Variable	B	SD	Wald *χ* ^2^	*p*	OR	95% CI
Intercept	−4.742	1.126	17.734	**< 0.001**	0.009	0.001–0.079
Follow‐up						
Baseline	0^a^	—	—	—	1.000	—
6‐month follow‐up	0.141	0.425	0.110	0.740	1.152	0.501–2.650
12‐month follow‐up	0.113	0.446	0.065	0.799	1.120	0.468–2.683
Age						
Age < 60	0^a^	—	—	—	1.000	—
Age ≥ 60	0.745	0.420	3.150	0.076	2.106	0.925–4.792
Education						
Junior high school and below	0^a^	—	—	—	1.000	—
High school	0.327	0.394	0.690	0.406	1.387	0.641–3.001
Associate degree and above	1.144	0.342	11.200	**0.001**	3.141	1.607–6.140
Disease duration, year	0.072	0.034	4.512	**0.034**	1.074	1.006–1.148
UPDRS‐III	0.006	0.020	0.080	0.777	1.006	0.968–1.045
H‐Y stage						
2.0	0^a^	—	—	—	1.000	—
2.5	0.502	0.554	0.823	0.364	1.652	0.558–4.891
3.0	0.874	0.599	2.129	0.145	2.396	0.741–7.748
4.0	1.257	0.712	3.114	0.078	3.516	0.870–14.204
Subtype						
PIGD	0^a^	—	—	—	1.000	—
TD	0.041	0.350	0.014	0.907	1.042	0.525–2.069
Intermediate	−0.428	0.574	0.556	0.456	0.652	0.212–2.007
LEDD, mg/day	< 0.001	< 0.001	0.227	0.634	1.000	0.999–1.001
Hb, g/L						
Anemic	0^a^	—	—	—	1.000	—
Not anemic	1.743	0.630	7.667	**0.006**	5.716	1.664–19.633
NMSS domains						
NMSS domain 1: Cardiovascular	0.442	0.375	1.392	0.238	1.556	0.746–3.243
NMSS domain 2: Sleep/fatigue	−0.062	0.084	0.543	0.461	0.940	0.798–1.108
NMSS domain 3: Mood/cognition	−0.194	0.096	4.063	**0.044**	0.823	0.682–0.995
NMSS domain 4: Perceptual problems/hallucinations	0.247	0.487	0.256	0.613	1.280	0.492–2.326
NMSS domain 5: Attention/memory	−0.121	0.196	0.382	0.537	0.886	0.603–1.301
NMSS domain 6: Gastrointestinal	0.297	0.134	4.893	**0.027**	1.346	1.034–1.752
NMSS domain 7: Urinary	0.264	0.098	7.286	**0.007**	1.302	1.075–1.577
NMSS domain 8: Sexual function	−0.027	0.155	0.030	0.863	0.974	0.719–1.319
NMSS domain 9: Miscellaneous	−0.080	0.135	0.354	0.552	0.923	0.709–1.202
Interaction effect of UPDRS III with NMSS domains						
UPDRSIII * NMSS domain 1	−0.006	0.006	0.861	0.353	0.994	0.982–1.007
UPDRSIII * NMSS domain 2	0.001	0.001	0.193	0.660	1.001	0.998–1.003
UPDRSIII * NMSS domain 3	0.003	0.002	4.061	**0.044**	1.003	1.000–1.007
UPDRSIII * NMSS domain 4	−0.003	0.008	0.179	0.672	0.997	0.982–1.012
UPDRSIII * NMSS domain 5	0.002	0.004	0.493	0.483	1.002	0.996–1.009
UPDRSIII * NMSS domain 6	−0.005	0.002	4.089	**0.043**	0.995	0.991–1.000
UPDRSIII * NMSS domain 7	−0.004	0.002	4.557	**0.033**	0.996	0.993–1.000
UPDRSIII *NMSS domain 8	0.002	0.0024	0.415	0.519	1.002	0.997–1.006
UPDRSIII * NMSS domain 9	0.001	0.002	0.405	0.525	1.001	0.997–1.006

*Note:* The Quasi‐Likelihood under the Independence Model Criterion (QIC) and the Quasi‐Likelihood under the Independence Model Criterion with a Correction for Clustering (QICC) indicated that the model with the first‐order autocorrelated working correlation matrix structure yielded the optimal fitting performance. Bold values mean adjusted *p* < 0.05.

Abbreviations: 95% CI, 95% confidence interval; B, coefficient; Hb, hemoglobin; H‐Y stage, Hoehn–Yahr stage; LEDD, levodopa equivalent daily doses; NMSS, Nonmotor symptoms scale; OR, odds ratio; PIGD, postural instability/gait difficulty subtype; SD, standard deviation; TD, tremor‐dominant subtype; UPDRS‐III, unified Parkinson's disease rating scale part III; Wald *χ* ^2^, Wald Chi‐Square.

^a^ means reference.

Additionally, the interaction effect of MS with mood/cognition symptoms (Wald *χ* ^2^ = 4.061, *p* = 0.044) positively correlated with the occurrence of falls; the interaction effect of MS with gastrointestinal symptoms (Wald *χ* ^2^ = 4.089, *p* = 0.043) and urinary symptoms (Wald *χ* ^2^ = 4.557, *p* = 0.033) negatively correlated with the occurrence of falls.

## 4 Discussion

The study on 136 PD patients undergoing STN‐DBS revealed that preoperative falls occurred in 24.3%, slightly decreasing to 19.9% postoperatively. Indoor falls increased, and freezing of gait decreased significantly over the 12‐month postoperative period. Postoperative fallers had more severe NMS and differed significantly in age, residence, and education. Univariate and multivariate analyses identified significant fall predictors, including higher education, longer disease duration, the absence of anemia, and specific NMS domains (mood/cognition, gastrointestinal, and urinary symptoms). Interaction effects of MS with mood/cognition, gastrointestinal, and urinary symptoms were also correlated with falls.

A systematic review of 22 studies found that 60.5% (35%–90%) of PD patients experienced falls [9]. Our study observed fall rates of 24.3% before STN‐DBS, 19.9% after, and 43.4% with combined data. Although no significant difference in fall rates before and after STN‐DBS was noted, a downward trend was noted. A study of 331 PD patients similarly reported fall improvement in about one‐third of patients post‐STN‐DBS, particularly those with more severe symptoms, while those with milder symptoms remained stable or worsened [31]. Another retrospective study observed an increase in falls over time post‐STN‐DBS [22]. These mixed findings require further clarification of the details regarding falls. In this case, we included mixed‐methods studies that combine quantitative and qualitative approaches to illustrate changes in falls within a socio‐ecological scenario. While the overall fall rate did not decrease, falls occurred more frequently indoors postoperatively. This shift may result from increased indoor walking and falls among patients with lower daily activity or a reduction in outdoor falls among those with milder symptoms, or both. Additionally, freezing of gait, a major contributor to falls [32], improved significantly at 12 months. However, the causes of falls in the real world are far more complex. Considering the finding in this study that falls did not improve, we speculate that changes in the freezing of gait can only partially explain the changes in falls, and other factors, such as NMS, may also contribute to the incidence of falls.

This study indicated that all NMSS domains, excluding gastrointestinal symptoms, showed significant improvement after the STN‐DBS surgery compared to the baseline, similar to findings in our previous study, with potential reasons analyzed [21] and aligned with previous studies [33, 34]. However, a prior study by Arai et al. [35] concluded a long‐term improvement in PD patients' gastric emptying dysfunction, which is a key gastrointestinal symptom in PD. These conflicting findings suggest ongoing debate regarding the effects of STN‐DBS on gastrointestinal symptoms. A possible explanation for this discrepancy is that gastrointestinal dysfunction in PD is influenced not only by central nervous system (CNS) pathology but also by disturbances in the autonomic nervous system (ANS) and enteric nervous system (ENS). While STN‐DBS primarily modulates the basal ganglia‐thalamocortical circuits, its effects on the ANS—particularly in regulating the complex neural networks governing gastrointestinal motility—may be limited [36]. The ENS, often referred to as the “second brain” operates semi‐independently of the CNS, and its dysfunction in PD may not be directly affected by STN‐DBS.

Additionally, the presence of peripheral neurodegeneration may contribute to the persistence of gastrointestinal symptoms despite improvements in other NMS domains. Although many nonmotor symptoms (NMS) in PD involve autonomic dysfunction—including cardiovascular, urinary, and thermoregulatory symptoms—these functions may be more directly linked to central autonomic control centers within the brainstem and diencephalon, areas that might be secondarily modulated by STN‐DBS. For instance, prior studies have reported improvements in autonomic‐related symptoms such as orthostatic hypotension and urinary dysfunction post‐DBS, possibly due to changes in dopaminergic and nondopaminergic pathways involved in autonomic regulation [37]. In contrast, gastrointestinal function is regulated by a broader network that extends beyond central circuits to the peripheral nervous system, making it less responsive to STN‐DBS intervention.

While STN‐DBS effectively improves multiple nonmotor symptoms, its impact on falls remains complex. Mechanistically, STN‐DBS has been shown to enhance dynamic balance but impair postural control, including quiet stance, reactive, and anticipatory postural adjustments [37]. Some studies have reported an increased incidence of falls following surgery, and others suggest that DBS itself may contribute to fall occurrences [17, 38, 39]. Our findings are consistent with these observations, indicating that although STN‐DBS significantly improves most NMS domains, it does not provide a clear benefit for fall reduction.

Falls in PD result from a multifaceted interplay of risk factors, influenced by STN‐DBS, which complicates fall prediction and prevention, increasing the complexity of fall prediction and prevention. We aimed to identify fall risk factors through subgroup analysis comparing nonfalling and falling groups. Our study observed differences in urinary function at baseline, and in residence at 6 months, as well as in residence, education, sleep/fatigue, attention/memory, gastrointestinal, and urinary domains, and total NMSS score at 12 months. Moreover, we found that postoperative falls were more common in patients over 60, residing in urban areas, with higher education levels, and higher urinary NMSS domain scores at baseline. These factors align with those proposed by The National Parkinson Foundation's Falls Task Force, categorized as age‐related and PD‐specific risk factors [40]. Importantly, MS severity did not differ between nonfalling and falling groups at baseline, highlighting the potential influence of NMS and their growing impact on postoperative fall occurrences.

To date, there has been a dearth of studies examining factors contributing to falls post‐STN‐DBS surgery, and no specific NMS predictors have been identified [22, 31]. This gap significantly hampers efforts in fall prevention and management, particularly for patients undergoing STN‐DBS. This is the first study to systematically include NMS in exploring fall factors in STN‐DBS patients and use the GEE method to analyze their longitudinal association with falls. Results indicated that longer disease duration, higher education, absence of anemia, milder mood/cognitive issues, and more severe gastrointestinal and urinary symptoms were associated with increased fall risk in PD patients. Longer disease duration may lead to more severe symptoms and functional decline, thereby increasing fall risk [41], particularly due to worsening axial postural instability as PD progresses. Unexpectedly, higher education and absence of anemia were associated with increased fall risk. Both factors might be related to a more active lifestyle, greater community engagement, and consequently a higher likelihood of falling, as suggested by others [42]. Understanding these associations can guide the development of targeted interventions to reduce fall risk and improve postoperative outcomes for PD patients.

The GEE model revealed complex interactions between NMS and fall risk in PD patients. The main effects revealed that worsened mood/cognition was associated with a reduced fall risk. Notably, the mood/cognition domain (6 items) of NMSS focuses primarily on apathy, including motivation (2 items) and anhedonia (2 items), but also includes anxiety (1 item) and depression (1 item). Although studies have suggested a link between mood issues and falls, the results have been inconsistent [43, 44]. Mood disturbances can influence falls in PD patients in two ways. On one hand, severe mood issues can have a negative impact on MS and worsen gait, increasing fall risk. On the other hand, patients with apathy or affective symptoms may reduce daytime activity, leading to fewer fall opportunities, particularly when motor and gait disturbances are present. Conversely, gastrointestinal and urinary symptoms were associated with increased fall risk, likely for the reasons outlined below. First, both gastrointestinal and urinary symptoms can cause physical discomfort and instability [45], leading to a higher likelihood of falls during daily activities. Second, both symptoms increase toileting urgency, which is linked to a higher risk of night falls and daily falls during the journey between their bedside and the toilet [46, 47]. Notably, gastrointestinal symptoms in PD patients did not significantly improve after STN‐DBS, while these symptoms were significantly associated with the occurrence of falls. In contrast, STN‐DBS can improve urinary symptoms by reducing detrusor overactivity and increasing bladder capacity, thereby reducing falls [47, 48]. These findings highlight the need for tailored fall prevention programs for this group of PD patients and the incorporation of multidisciplinary treatments when STN‐DBS and dopaminergic therapy are insufficient.

The GEE model also observed interaction effects between MS and NMS on falls. As Parkinson's MS worsened, the protective effect of mood/cognition symptoms diminished or even reversed. Meanwhile, although gastrointestinal and urinary issues increase fall risk, effects were less pronounced when MS were more severe. This might be because severe MS overshadow the impact of gastrointestinal and urinary problems on fall risk, or patients with severe MS might have overall reduced activity levels, mitigating the effect of these symptoms on falls. The interaction effects showed that the relationship between nonmotor symptoms and fall risk is complex and depends on the severity of motor symptoms. The reversal of mood/cognition's protective effect in advanced motor stages may reflect two interrelated phenomena:

1. Threshold Dominance of Motor Disability: In severe PD, profound postural instability, freezing of gait, and bradykinesia dominate fall risk, overshadowing contributions from mood/cognition. Patients may become less exposed to fall‐triggering scenarios (e.g., multitasking, ambulation in complex environments) due to immobility, masking the typical risk posed by apathy or executive dysfunction.
2. Neurodegenerative Overlap: Mood/cognitive deficits in advanced PD often correlate with diffuse Lewy body pathology in limbic and cortical regions. This progression may disrupt compensatory mechanisms (e.g., attention to balance), eroding any residual protective effects seen in earlier stages.

The attenuation of gastrointestinal/urinary effects in severe motor disease could stem from reduced physical activity levels (limiting opportunities for falls triggered by urgency or orthostasis) or caregiver supervision, which mitigates risks despite persistent symptoms. Understanding these interactions can help tailor more effective fall prevention strategies considering both MS and NMS.

Several limitations should be acknowledged. First, this study was conducted at a single center, which may introduce selection bias. Second, the sample size and the follow‐up time were relatively limited, which might lead to the limited generalizability of findings. Thus, multicenter studies with larger sample sizes and longer follow‐up observations are further needed. Third, fall events were self‐reported by patients, which may have introduced recall bias. Patients with cognitive impairments or fluctuating awareness might underreport or inaccurately recall fall incidents, leading to potential misclassification. To improve data accuracy in future studies, integrating objective fall‐monitoring tools such as wearable sensors, accelerometers, or caregiver‐reported fall logs could provide a more reliable assessment of fall frequency and severity. Fourth, due to the necessity of conducting UPDRS‐III assessments in a hospital setting, the follow‐up rate for these assessments post‐STN‐DBS was low. This limitation restricted our ability to analyze the influence of motor symptom changes on fall risk comprehensively. Future studies should explore alternative follow‐up strategies, such as telemedicine‐based assessments or home‐based wearable motor evaluations, to improve data collection on motor symptom progression and its relationship with fall risk. Lastly, several key confounding variables, including axial function impairments, cognitive abilities, and the severity of freezing of gait, were not considered due to data accessibility constraints. These factors are known to significantly influence fall risk in PD patients. Subsequent research should prioritize comprehensive data collection on these variables, possibly integrating multimodal assessments such as neuropsychological testing, gait analysis, and machine learning‐based predictive models to better understand the multifactorial nature of falls in PD patients post‐DBS.

## 5 Conclusions

Our study found significant improvements in all NMSS domains with the exception of gastrointestinal. Despite these improvements, the overall fall rate did not show a significant decrease following surgery. NMS, including mood/cognition, gastrointestinal, and urinary symptoms, are associated with falls, and their interaction with MS also affects fall risk. These findings highlight the intricate relationship between NMS and fall risk, emphasizing the need for a comprehensive fall prevention strategy that incorporates targeted interventions for NMS.

## Author Contributions

Ying Gao contributed to study design, data collection, data analysis, and manuscript writing. Hui You contributed to study design, data analysis, and manuscript revision. Jue Wang was involved in data collection and literature review. Mengsi Yao contributed to data collection and literature review. Dianyou Li contributed to clinical insights and study design. Bomin Sun was involved in study supervision and study design. Linbin Wang contributed to study design, result interpretation, and manuscript revision. Xian Qiu was involved in project supervision, study design, result interpretation, and manuscript revision.

## Ethics Statement

This study was approved by the Ethics Committee of Ruijin Hospital (Ruijin Hospital Ethics Committee [2023] NO. 122), and written informed consent was obtained from all participating patients.

## Consent

The authors agree to publication in the journal.

## Conflicts of Interest

The authors declare no conflicts of interest.

## Supporting information


Data S1

## Data Availability

The data that support the findings of this study are available on request from the corresponding author. The data are not publicly available due to privacy or ethical restrictions.
