# Supplementary material for: Nonmotor Symptom Changes and Their Association With Falls Among Parkinson's Disease Patients Undergoing Deep Brain Stimulation: A 1‐Year Cohort Study
Source: CNS Neurosci Ther. 2025 Feb 28;31(3):e70310. doi: 10.1111/cns.70310 (PMC11871399; doi:10.1111/cns.70310)
Supplement: Supplementary file 1 — Data S1 [file CNS-31-e70310-s001.docx]

**Supplementary Materials**

**Parkinson’s disease patient fall questionnaire**

1. Have you experienced any falls in the past month?

(1) No; (2) Yes (If yes, please specify how many times you have fallen.)

2. Where did the fall occur? (You may select more than one option.)

(1) Indoors; (2) Outdoors

3. What were you doing at the time of the fall? (You may select more than one option.)

(1) Walking; (2) Standing or still; (3) Transitioning between activities

4. Were you performing multiple tasks at the time of the fall?

(1) No, I was doing only one task; (2) Yes, I was multitasking

5. When did the fall occur? (You may select more than one option.)

(1) Morning; (2) Noon; (3) Afternoon; (4) Before bedtime; (5) Midnight

6. Did you experience freezing of gait?

(1) Yes; (2) No

7. Did you have any warning symptoms (e.g. light-headedness, vertigo, loss of consciousness) before the fall?

(1) Yes; (2) No

8. Were you alone during the fall?

(1) Yes; (2) No

9. Did you experience orthostatic hypotension?

(1) Yes; (2) No

10. Were you in an “off” period of your medication?

(1) Yes; (2) No

11. Are you taking any sleep medications?

(1) Yes; (2) No

12. After the fall, did you experience:

(1) Serious injuries such as fractures; (2) Minor injuries such as abrasions; (3) None

**Supplementary Table 1**. Demographic and disease information (*N* = 136).

| **Variable** | **Mean (SD) / n (%)** |
| --- | --- |
| **Age** | 62.8 (9.5) |
| **Gender** |  |
| Male | 80 (58.8) |
| Female | 56 (41.2) |
| **Residence** |  |
| Rural area | 26 (19.1) |
| Urban area | 110 (80.9) |
| **Occupation** |  |
| Retired | 103 (75.7) |
| On duty | 19 (14.0) |
| Unemployed | 14 (10.3) |
| **Marriage** |  |
| Married | 134 (98.5) |
| Unmarried/Divorced/Widowed | 2 (1.5) |
| **Education** |  |
| Junior high school and below | 61 (44.9) |
| High school | 36 (26.5) |
| Associate degree and above | 39 (28.7) |
| **Disease duration, years** | 8.0 (5.0) ^a^ |
| **LEDD, mg/day** | 700.0 (426.0) ^a^ |
| **UPDRS-III** | 57.8 (13.2) |
| **H-Y stage** |  |
| 2.0 | 17 (12.5) |
| 2.5 | 44 (32.4) |
| 3.0 | 59 (43.4) |
| 4.0 | 16 (11.8) |
| **Subtype** |  |
| PIGD | 85 (62.5) |
| TD | 36 (26.5) |
| Intermediate | 15 (11.0) |
| **With comorbidity** |  |
| No | 100 (73.5) |
| Yes | 36 (26.5) |
| **BMI, kg/m^2^** | 23.7 (3.1) |
| **Hb, g/L** | 137.7 (13.6) |

*Abbreviation: LEDD, Levodopa equivalent daily doses; UPDRS-III, Unified Parkinson’s disease rating scale part III; H-Y stage, Hoehn-Yahr stage; PIGD, Postural instability/gait difficulty subtype; TD, Tremor-dominant subtype; BMI, Body mass index; Hb, Hemoglobin.*

*All the information was collected during “off” conditions of patients.*

*a. Data are presented as median (IQR).*

**Supplementary Table 2.** Analysis for the specifics of falls in PD patients preoperatively, 6 months postoperatively, and 12 months postoperatively to compare the differences.

|  | **Baseline** | **6-month follow-up** | **12-month follow-up** | ***Z/χ^2^*** | ***P*** |
| --- | --- | --- | --- | --- | --- |
| **Fall count, Mean (SD)** | 0.5 (1.3) | 0.4 (1.0) | 0.5 (1.1) | 1.565 | 0.447 |
| **Fall location** |  |  |  | 15.883 | **0.001** |
| Indoors | 21.4% | 85.8% | 77.3% |  |  |
| Outdoors | 42.9% | 7.1% | 4.5% |  |  |
| Both | 35.7% | 7.1% | 18.2% |  |  |
| **Fall status** |  |  |  | 8.423 | 0.067 |
| Walking or moving | 92.9% | 50.0% | 50.0% |  |  |
| Standing or still | 7.1% | 28.6% | 22.7% |  |  |
| Both | 0.0% | 21.4% | 27.3% |  |  |
| **Number of tasks at the time of the fall** |  |  |  | 3.586 | 0.149 |
| Single task | 85.7% | 100.0% | 100.0% |  |  |
| Multitask | 14.3% | 0.0% | 0.0% |  |  |
| **Fall time** |  |  |  | 2.296 | 0.790 |
| Daytime | 85.7% | 78.6% | 77.3% |  |  |
| Nighttime | 0.0% | 14.3% | 9.1% |  |  |
| Both | 14.3% | 7.1% | 13.6% |  |  |
| **Freezing gait** |  |  |  | 9.487 | **0.008** |
| Yes | 57.1% | 28.6% | 9.1% |  |  |
| No | 42.9% | 71.4% | 90.9% |  |  |
| **Precursor symptom** |  |  |  | 2.960 | 0.249 |
| Yes | 42.9% | 14.3% | 22.7% |  |  |
| No | 57.1% | 85.7% | 77.3% |  |  |
| **Alone at the time of the fall** |  |  |  | 1.870 | 0.419 |
| Yes | 71.4% | 50.0% | 50.0% |  |  |
| No | 28.6% | 50.0% | 50.0% |  |  |
| **Postural hypotension** |  |  |  | 2.180 | 0.450 |
| Yes | 85.7% | 0.0% | 4.5% |  |  |
| No | 14.3% | 0.0% | 95.5% |  |  |
| **Fall during a drug “off” period** |  |  |  | 5.598 | 0.068 |
| Yes | 42.9% | 28.6% | 9.1% |  |  |
| No | 57.1% | 71.4% | 90.9% |  |  |
| **Take sleep aids** |  |  |  | 0.691 | 0.832 |
| Yes | 21.4% | 28.6% | 18.2% |  |  |
| No | 78.6% | 71.4% | 81.8% |  |  |
| **Fall outcomes** |  |  |  | 4.182 | 0.377 |
| Serious injuries such as fractures | 14.3% | 0.0% | 9.1% |  |  |
| Minor injuries such as abrasions | 35.7% | 28.6% | 13.6% |  |  |
| None | 50.0% | 71.4% | 77.3% |  |  |

*Bold values mean p < 0.05.*


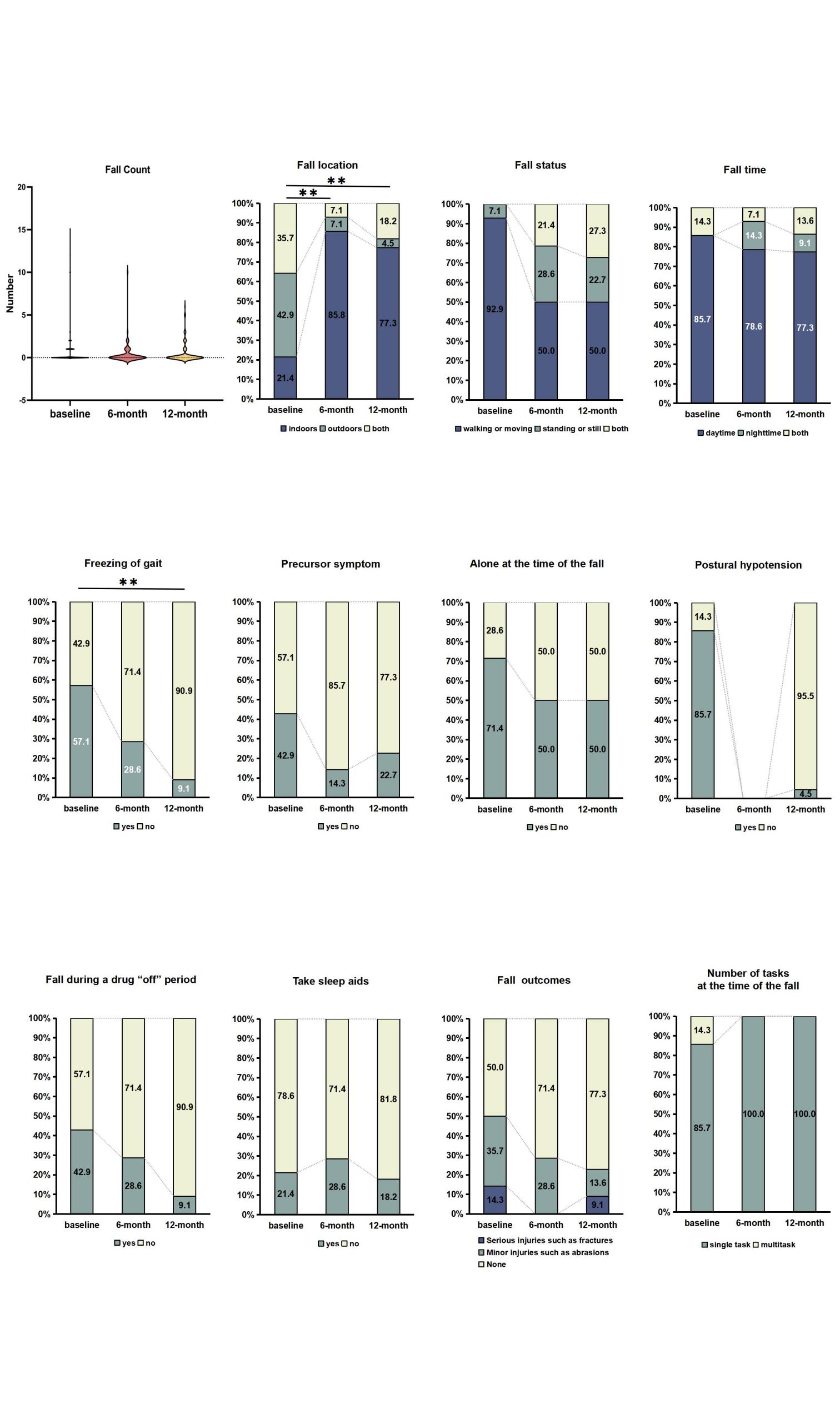


**Supplementary Figure 1.** Percentage accumulation charts showing chi-square test results for the specifics of falls in PD patients preoperatively, 6 months postoperatively, and 12 months postoperatively to compare the differences (*** means P < 0.01*).

**Supplementary Table 3.** Univariate generalized estimating equation analysis of factors to falls after DBS in PD patients.

| **Variable** | **B** | **SD** | **Wald *χ^2^*** | ***P*** | **OR** | **95%CI** |
| --- | --- | --- | --- | --- | --- | --- |
| **Follow-up** |  |  |  |  |  |  |
| Baseline | 0^a^ | - | - | - | 1.000 | - |
| 6-month follow-up | -0.257 | 0.264 | 0.949 | 0.330 | 0.773 | 0.461-1.297 |
| 12-month follow-up | -0.257 | 0.284 | 0.819 | 0.366 | 0.773 | 0.443-1.350 |
| **Age** |  |  |  |  |  |  |
| Age＜60 | 0^a^ | - | - | - | 1.000 | - |
| Age≥60 | 0.889 | 0.356 | 6.229 | **0.013** | 2.434 | 1.210-4.894 |
| **Gender** |  |  |  |  |  |  |
| Male | 0^a^ | - | - | - | 1.000 | - |
| Female | 0.011 | 0.285 | 0.001 | 0.970 | 1.011 | 0.578-1.766 |
| **Residence** |  |  |  |  |  |  |
| Rural areas | 0^a^ | - | - | - | 1.000 | - |
| Urban areas | 0.864 | 0.459 | 3.543 | 0.060 | 2.373 | 0.965-5.836 |
| **Education** |  |  |  |  |  |  |
| Junior high school and below | 0^a^ | - | - | - | 1.000 | - |
| High school | 0.266 | 0.346 | 0.589 | 0.443 | 1.305 | 0.662-2.572 |
| Associate degree and above | 0.778 | 0.340 | 5.234 | **0.022** | 2.177 | 1.118-4.239 |
| **Disease duration, year** | 0.077 | 0.028 | 7.750 | **0.005** | 1.080 | 1.023-1.140 |
| **UPDRS-III** | 0.015 | 0.011 | 1.975 | 0.160 | 1.015 | 0.994-1.037 |
| **H-Y** |  |  |  |  |  |  |
| 2.0 | 0^a^ | - | - | - | 1.000 | - |
| 2.5 | 0.459 | 0.456 | 1.014 | 0.314 | 1.583 | 0.648-3.867 |
| 3.0 | 0.816 | 0.424 | 3.711 | 0.054 | 2.261 | 0.986-5.185 |
| 4.0 | 1.414 | 0.573 | 6.082 | **0.014** | 4.113 | 1.337-12.654 |
| **Subtype** |  |  |  |  |  |  |
| PIGD | 0^a^ | - | - | - | 1.000 | - |
| TD | -0.243 | 0.343 | 0.501 | 0.479 | 0.784 | 0.400-1.537 |
| Intermediate | -0.693 | 0.488 | 2.016 | 0.156 | 0.500 | 0.192-1.302 |
| **With comorbidity** |  |  |  |  |  |  |
| No | 0^a^ | - | - | - | 1.000 | - |
| Yes | 0.357 | 0.304 | 1.386 | 0.239 | 1.430 | 0.788-2.592 |
| **LEDD, mg/day** | 0.001 | ＜0.001 | 4.772 | **0.029** | 1.001 | 1.000-1.001 |
| **BMI, kg/m^2^** |  |  |  |  |  |  |
| BMI＜18.5 | 0^a^ | - | - | - | 1.000 | - |
| 18.5≤BMI＜24.0 | 0.462 | 0.303 | 2.327 | 0.127 | 1.588 | 0.877-2.876 |
| BMI≥24.0 | 0.916 | 0.522 | 3.082 | 0.079 | 2.500 | 0.899-6.954 |
| **Hb, g/L** |  |  |  |  |  |  |
| Anemic | 0^a^ | - | - | - | 1.000 | - |
| Not anemic | 1.801 | 0.488 | 13.608 | **＜0.001** | 6.058 | 2.326-15.775 |
| **NMSS domains** |  |  |  |  |  |  |
| NMSS domain 1: Cardiovascular | 0.160 | 0.071 | 5.005 | **0.025** | 1.173 | 1.020-1.349 |
| NMSS domain 2: Sleep/fatigue | 0.024 | 0.012 | 4.071 | **0.044** | 1.024 | 1.001-1.049 |
| NMSS domain 3_:_ Mood/cognition | 0.028 | 0.011 | 6.813 | **0.009** | 1.029 | 1.007-1.051 |
| NMSS domain 4: Perceptual problems/hallucinations | 0.125 | 0.051 | 5.957 | **0.015** | 1.133 | 1.025-1.252 |
| NMSS domain 5: Attention/memory | 0.060 | 0.030 | 3.937 | **0.047** | 1.062 | 1.001-1.126 |
| NMSS domain 6: Gastrointestinal | 0.051 | 0.020 | 6.478 | **0.011** | 1.053 | 1.012-1.095 |
| NMSS domain 7: Urinary | 0.045 | 0.014 | 10.888 | **0.001** | 1.046 | 1.019-1.075 |
| NMSS domain 8: Sexual function | 0.061 | 0.032 | 3.617 | 0.057 | 1.063 | 0.998-1.131 |
| NMSS domain 9: Miscellaneous | 0.028 | 0.015 | 3.743 | 0.053 | 1.028 | 1.000-1.058 |

*Abbreviation: B, Coefficient; SD, Standard deviation; Wald χ²: Wald Chi-Square; OR: Odds ratio; 95% CI: 95% Confidence interval; UPDRS-III, Unified parkinson’s disease rating scale part III; H-Y stage, Hoehn-Yahr stage; PIGD, Postural instability/gait difficulty subtype; TD, Tremor-dominant subtype; LEDD, Levodopa equivalent daily doses; Hb, Hemoglobin; NMSS: Non-motor symptoms scale.*

*Bold values mean P < 0.05.*
